# Supplementary material for: Nurses’ work-related stress and associated factors in governmental hospitals in Harar, Eastern Ethiopia: A cross-sectional study
Source: PLoS One. 2020 Aug 3;15(8):e0236782. doi: 10.1371/journal.pone.0236782 (PMC7398531; doi:10.1371/journal.pone.0236782)
Supplement: Attachment — Submitted filename: Response to Reviewers.docx [file pone.0236782.s001.docx]

Response to Reviewers’

First of all, we would like to appreciate the PLOSE ONE Journal Academic Editor and Reviewers for providing us timely and constructive comments to revise our manuscript that suits the PLOS ONE Journal standards and requirements. We are also pleased to submit the revision that responds to each point raised by the academic editor/ reviewers and presented as follows.

1. Please ensure that your manuscript meets PLOS ONE's style requirements, including those for file naming.

- Upon our revision, we have seen that some of the manuscript parts do not meet the PLOS ONE's style requirements. After thoroughly reading the PLOS ONE's submission guidelines we made changes that meet the requirement.

2. We suggest you thoroughly copyedit your manuscript for language usage, spelling, and grammar.

- As much as possible we tried to improve the manuscript for language usage, spelling, and grammar by consulting our colleagues and staff language professionals.

3. We noticed you have some minor occurrence of overlapping text with the following previous publication(s), which needs to be addressed:

<https://www.researchgate.net/publication/264888293_Effect_Procrastination_on_Work-Related_Stress>

<http://www.imedpub.com/articles/occupational-stress-management-among-nurses-in-selected-hospital-in-benin-city-edo-state-nigeria.php?aid=12016>

<https://www.scirp.org/journal/PaperInformation.aspx?PaperID=91107>

- Thanks for your link and we searched and have seen some minor occurrence of overlapping text especially with the last two papers. The overlapping was on the definition of “stress” and we quoted for that and rephrased the rest text.

4. Please include additional information regarding the survey or questionnaire used in the study and ensure that you have provided sufficient details that others could replicate the analyses.

- As mentioned in the methodology part of the manuscript, the tool (questionnaire) is a structured and pre-tested self-administered questionnaire. The tool consisted of the socio-demographics and ques­tions related to the work environment, substance use, illness, and stress.
- Work-related stress was assessed based on the Expanded Nursing Stress Scale (ENSS). ENSS is an expanded and updated Nursing Stress Scale developed by French SE, Lenton R, Walters V, and Eyles J. The ENSS was obtained directly from the author Susan French communicating through email ([susan.french@mcgill.ca](mailto:susan.french@mcgill.ca)) and getting permission.
- The Expanded Nursing Stress Scale contained 57 items in nine subscales: death and dying (7 items); conflict with physicians (5 items); inadequate emotional preparation (3 items); problems relating to peers (6 items); problems relating to supervisors (7 items); workload (9 items); patients and their families (8 items); discrimination (3 items) and treatment uncertainty (9 items). Participants were asked to indicate the frequency of work-related stress using a 4-point Likert response scale. Response options were ‘never stressful’ (1), ‘occasionally stressful’ (2), ‘frequently stressful’ (3) and ‘always stressful’ (4). The category “not applicable” was scored as zero (0).
- In order to compute the total stress score, we added together with the scores on all 57 items. In order to measure scores on specific subscales, the appropriate items should be added together and the higher the score, the greater the frequency of stress on any subscale. The higher the score, the more the respondent agrees that the situation was stressful.
- Work-related stress is determined by computing the mean of all 57 items for every study participant.
- In this study, the mean values 2 and above were assumed to indicate Work-related stress.
- Based on a study done on a random sample of 2,280 nurses in Ontario, Canda, the authors demonstrated the ENSS reliability with Cronbach’s alpha (α = 0.96) and the individual subscale reliability ranged from α = 0.88 (problems with supervisors) to α = 0.65 (discrimination) [31].
- In the current study, Cronbach’s alpha coefficient was found to be 0.86 for the overall scales [**Table1**]**.**

5. We note that you have provided funding information that is not currently declared in your Funding Statement. However, funding information should not appear in the Acknowledgments section or other areas of your manuscript. We will only publish funding information present in the Funding Statement section of the online submission form.

- We removed funding information in our revised manuscript and only included in the Funding Statement section of the online submission form.

6. The introduction is too brief; there is a need for a thorough review of literature to indicate the problem.

- In the revised manuscript we have included the prevalence and effects of work-related stress on the performance of the organization as well as the nurses in Ethiopia and other countries. We also incorporated the justification of conducting this study.
- We revised the methodology part and made some clarification.
- We also incorporated and compared similar studies conducted in Ethiopia and other parts of Africa.

7. The total number of governmental hospitals in the study area (in the Harar region) and the selection of hospitals is not clear?

- There are a total of four governmental hospitals (2 military and 2 public hospitals) in the Harari region. Namely, the 2 military hospitals are Police Hospital (PH) and Army Hospital (AH). Likewise, the 2 public hospitals are Haramaya University Specialized Teaching Hospital (HUSTH) and Jugel Hospital (JH). The military hospitals usually serve the police and army population, on the other hand, the public hospitals serve the general population.
- The study was conducted in the four Governmental hospitals found in the Harari region. There were a total of 446 nurses working in these four Governmental hospitals during the study period. Out of these, HUSTH comprised 244 nurses and JH had 97 nurses. Similarly, there were 55 nurses in AH and 50 nurses in PH working in different departments.
- The sample size was calculated using a single population proportion formula at 95% significance level, by considering the proportion of work-related stress (37.8%) in Addis Ababa with 5% margin of error, and adding 10% non-response rate. Therefore, the total sample size was 398 nurses.
- A stratified sampling technique was applied considering the difference in the level of work-related stress across hospitals. Therefore, the total sample size was proportionally allocated to the size of the four government hospitals so that HUSTH, JH, AH and PH constituted 217, 87, 49 and 45 participants respectively. The sampling frame was constructed for each hospital separately by taking the nurses list from the human resource management department of each hospital. Then, study participants were selected from respective hospital units using a simple random sampling technique.

8. What are the inclusion and exclusion criteria of the study? Not clearly mentioned or I think it is missed from the manuscript?

- Of course, we have seen that we missed the inclusion and exclusion criteria of the study in our submitted manuscript so that we incorporated in the revised manuscript.
- **Inclusion and exclusion criteria of the study**
- Inclusion Criteria
- All nurses who were fulltime employees of Harari regional state governmental hospitals were included.
- Exclusion Criteria
- Nurses who were seriously ill or unable to give responses due to this illness during the data collection period were excluded.

9. What is your assumption with the outcome variables from the study units or across hospitals is it similar or different?

- We assumed that the level of work-related stress across each hospital is different due to the difference in the population they served so that a stratified random sampling technique was used to select study participants from each hospital to maintain proportionality among each hospital.

10. Variable measurement or operational definition Missed.

- Operational Definitions:
- **Work-related stress**: is defined as the physical and emotional reactions that occur when the nurses' abilities and resources imbalance with the demands and requests of their work.
- **Nurses' work-related stress**: was rated from 1 (never stressful) to 4 (always stressful); the mean score of 2 or higher of 57 items was considered as work-related stress.
- **Substance use**: use or consumption of any substance such as alcohol, chat, cigarettes, shisha, and hashish, regardless of the amount and frequency of use for the past 3 months.
- **Job satisfaction**: Job Satisfaction was defined as whether respondents like (satisfied) or dislike (dissatisfied) their jobs. In this study, nurses who a­swered “yes” were assumed to be satisfied with their job.
- **Chronic medical illness**: ‘Chronic medical disease’ was categorized as ‘yes’ if hypertension, cardiovascular disease, AIDS, diabetes mellitus or arthritis had been diagnosed.

11. What is Cronbach’s alpha coefficient implications and its cut off point for conclusion?

- Cronbach’s alpha coefficient is a measure of internal consistency.
- It is considered to be a measure of scale reliability.
- A reliability coefficient of 0.70 or higher is considered acceptable for conclusion.

12. Was the tools used for the study were, the primary justifications for variations of different findings with yours, what are other possible expectations or perspectives behind for the difference can you suggest?

- In fact, the cause of stress in an individual person is difficult to determine since the stressors may vary from culture to culture even nurses working in different hospital units exposed to different types of work-related stress. Therefore, the variations for the level of stress among study findings might be due to the difference in the working environment itself or other factors in addition to the tools used to measure the stress level. So, further qualitative research could be used to explore and describe the experiences of stress among nurses in the work environment.

13. Making the general conclusion with the descriptive result is not recommended.so try to remove this sentence "The descriptive analysis showed that the workload was the most frequently stressful sub-scale.”

- We accepted it and removed it in the revised manuscript.

14. The manuscript has no page numbers. The editor relies on whole article pagination as downloaded from the website. Assuming that the Abstract is on page 8.

- Sorry for the omission unintentionally and we have inserted page numbers and line numbers for each page of the manuscript.

15. Please include socio-demographics in abstract results.

- We accepted and did it in the revised manuscript.

16. The sampling section was not clearly written. Please revisit. The author starts with simple random sampling but ends up with stratified random sampling but provides numbers for each stratum. The sampling frame is small (446). Why was a census not considered?

- We have indicated how to select study samples in question number 7 of this letter.
- It could be sounder enough if we had applied census for the study but we did not have adequate resources and time to apply census during the study period and we also considered that the sample size is acceptable for this study.

17. Keep reporting of numbers to 1 decimal place.

- We accepted and did it in the revised manuscript.
