# Supplementary material for: Nurses’ work-related stress and associated factors in governmental hospitals in Harar, Eastern Ethiopia: A cross-sectional study
Source: PLoS One. 2020 Aug 3;15(8):e0236782. doi: 10.1371/journal.pone.0236782 (PMC7398531; doi:10.1371/journal.pone.0236782)
Supplement: Attachment — Submitted filename: Response to Reviewers.docx [file pone.0236782.s002.docx]

Response to Reviewers’

First of all, we would like to appreciate the PLOSE ONE Journal Academic Editor and Reviewers for providing us timely and constructive comments to revise our manuscript that suits the PLOS ONE Journal standards and requirements. We are also pleased to submit the revision that responds to each point raised by the academic editor/ reviewers and presented as follows.

1. Please correct the sampling technique uses, in the abstract, you stated that simple random sampling was used and in the method section, you mention stratified random sampling.

- As you mentioned, we have seen that stratified random sampling was reported in the methods section and simple random sampling both in the abstract and in the methods section. A stratified sampling technique was used to proportionally allocate the number of participants to be involved in the study by considering the difference in the level of work-related stress across hospitals. Then we used a simple random sampling technique to select study participants from each hospital.

2. The study population was 442 and the sample 398, I do not understand how simple random sampling was used when almost all the nurses were included in the study. How did you randomly select 217 from 244 nurses, 96 from 97 nurses and 50 from 55 nurses? The appropriate sampling technique for the study would be a census were all the nurses were eligible to participate. Provide details or change the sampling to a census and clarify how the participants were recruited to the study.

- The study population was **446** and the sample **398**. Out of 446 study population, HUSTH comprised **244** nurses and JH had **97** nurses. Similarly, there were **55** nurses in AH and **50** nurses in PH. As we have indicated in the methods section, the total sample size was proportionally allocated to the size of the four government hospitals so that HUSTH, JH, AH and PH constituted **217**, **87**, **49** and **45** participants respectively. Then, the sampling frame was constructed for each hospital separately by taking the nurses list from the human resource management department of each hospital and study participants were selected from respective hospitals using a simple random sampling technique.
- In fact, It could be sounder enough if we had applied census for the study but we did not have adequate resources and time to apply census during the study period and we also considered that the sample size was acceptable for this study.

3. Delete the subheading “Inclusion and exclusion criteria” and integrate the description of the population under study population

- We accepted and corrected it in the revised manuscript.

4. Figure,“distribution of study participants by the hospital, is not necessary, delete and present the data in the narrative

- We accepted and corrected it in the revised manuscript.

5. Please explain child rearing

- **Child-rearing**: Child rearing was categorized as ‘yes’ if the person is parenting or taking care of a child/children
- We also incorporated it under operational definition of variables in the revised manuscript.

6. Make this the second statement after the prevalence. The descriptive analysis indicated that the workload was the most 298 stressful subscale for nurses. Tell us why this is the case and the implications of the findings before telling us that- this was consistent with other studies in different countries [(2717, 299 36-4624-34]). This might be due to a shortage of staff, extra non-nursing tasks and less time to support each other emotionally-support this statement with literature. Only then can you compare the prevalence with other studies. Please be brief in your comparison and group all the studies that are similar then those that are in contrast.

- We accepted and corrected it in the revised manuscript.

7. The implication should be part of the conclusion and not a subheading

- We accepted and corrected it in the revised manuscript.

8. Child-rearing is not discussed but appears in the conclusion

- We have seen and corrected it in the revised manuscript.

9. There are still major grammar, tenses, and punctuation errors-the manuscript should be copy edited by a professional English language editor. Please provide proof with your re-submission.

- We tried to improve the manuscript for grammar, tenses, and punctuation errors by consulting our colleagues and staff language professionals.
