# Supplementary material for: Nurses’ work-related stress and associated factors in governmental hospitals in Harar, Eastern Ethiopia: A cross-sectional study
Source: PLoS One. 2020 Aug 3;15(8):e0236782. doi: 10.1371/journal.pone.0236782 (PMC7398531; doi:10.1371/journal.pone.0236782)
Supplement: Attachment — Submitted filename: Response to Reviewers.docx [file pone.0236782.s003.docx]

Response to Reviewers’

First of all, we would like to appreciate the PLOSE ONE Journal Academic Editor and Reviewers for providing us timely and constructive comments to revise our manuscript that suits the PLOS ONE Journal standards and requirements. We are also pleased to submit the revision that responds to each point raised by the academic editor/ reviewers and presented as follows.

1. The sampling procedure and its validity, that remains pending to be clarified.

- The calculated sample size (398) was proportionally allocated to four government hospitals (HUSTH, JH, AH, and PH) as indicated in the methods section of the revised manuscript.
- Then, the sampling frame was constructed for each hospital separately by taking the nurses list from the human resource management department of each hospital and study participants were selected from respective hospitals using **simple random sampling technique**.
- We have reported what we have done in the study.we considered that the sampling procedure followed acceptable scientific standards and the sample size was adequate for this study.

2. The English writing of the paper, that needs additional revisions.

- We made extensive revisions to the revised manuscript to improve grammar, tenses, and punctuation errors by consulting our colleagues and staff English language professionals.
